# Comparative Evaluation of Mechanical Behaviour and Machinability of WAAM-Fabricated Aluminium Alloys

**DOI:** 10.3390/ma19122653

**Published:** 2026-06-20

**Authors:** Saravanamurugan Sundaram, Thenarasu Mohanavelu, Sumesh Arangot, Jana Petru, Mohan Ruthramoorthy, Rashaad Sabir Rowther, Kamalesh Senthilkumar

**Affiliations:** 1 Department of Mechanical Engineering, Amrita School of Engineering, Amrita Vishwa Vidyapeetham, Coimbatore 641112, India; s_saravana@cb.amrita.edu (S.S.); a_sumesh@cb.amrita.edu (S.A.); cb.en.u4mee23024@cb.students.amrita.edu (M.R.); cb.en.u4mee23033@cb.students.amrita.edu (R.S.R.); cb.en.u4mee23023@cb.students.amrita.edu (K.S.); 2 Department of Machining, Assembly and Engineering Metrology, Faculty of Mechanical Engineering, VSB-Technical University of Ostrava, 70800 Ostrava, Czech Republic

**Keywords:** Wire Arc Additive Manufacturing (WAAM), thin-walled aluminium alloy, machinability, power spectral density, porosity, chatter

## Abstract

This study presents an integrated evaluation of the mechanical properties and machinability of thin-walled aluminium alloys fabricated via Wire Arc Additive Manufacturing (WAAM), comparing them against conventional wrought counterparts. Experimental investigations were conducted through tensile testing, hardness measurement, surface characterisation, and cutting force analysis. The results reveal a critical performance trade-off: WAAM specimens demonstrated superior bulk mechanical properties, most notably a 44.36% increase in tensile strength, alongside enhanced elongation despite a marginal reduction in hardness. However, this structural advantage is counterbalanced by a significant machinability penalty. Frequency domain analysis using Power Spectral Density (PSD) revealed that inherent microstructural porosity in WAAM components triggers dynamic instabilities during machining. These irregularities make the material highly susceptible to high-frequency chatter, ultimately resulting in a 46.73% increase in surface roughness. By establishing a direct relationship between fabrication-induced microstructural defects and dynamic machining degradation, these findings emphasise the necessity for defect-aware, optimised hybrid manufacturing strategies to improve the industrial applicability of WAAM-fabricated structures.

## 1. Introduction

### 1.1. WAAM Fabrication and Mechanical Characteristics of Thin-Walled Aluminium Alloys

Thin-walled aluminium structures are extensively employed in aerospace, automotive, marine, and transportation industries owing to their excellent strength-to-weight ratio, corrosion resistance, and favourable thermal and electrical properties [1]. Manufacturers are now studying additive manufacturing (AM) techniques to fabricate thin-walled aluminium components because of their high material efficiency, high complexity and near-net-shaped part-producing capabilities [2]. Wire Arc Additive Manufacturing (WAAM) is one of the additive manufacturing techniques, and it utilises welding processes like GTAW, GMAW, etc., to fabricate the required part in layers by depositing the molten wire of an alloy or a metal on a specific base plate made from a compatible material. WAAM is known for its higher deposition rates, cost effectiveness, energy efficiency and material efficiency. The deposition rate of metal wire in the WAAM process is around 15–130 g/min, which is higher than the conventional additive manufacturing process [3]. Material cost is reduced to 7–69% with the WAAM technique. The reduction in fabrication time is around 40–60% and the reduction in post-machining time is around 15–20% [4]. Though WAAM is used to fabricate near-net-shaped parts, the surface finish is poor, demanding finish machining operations for better surface quality.

Residual stress, porosity and mechanical anisotropy are some of the major defects observed in WAAM specimens [5]. These defects can be mitigated by employing techniques like interpass cooling and cold rolling of WAAM deposited layers [6]. WAAM-fabricated Al alloys included porosity, and its presence was associated with the presence of moisture and contaminants in the shielding gas employed [7]. The presence of porosity was associated with the turbulence caused due to higher shielding gas flow rates [8]. Oxidation was observed in WAAM-fabricated Al alloy when the oxygen concentration exceeded 100 ppm in the process zone, though higher gas flow rates and closer nozzle distances improved shielding and decreased oxidation [9]. Higher travel speeds solve several problems, like reducing the risk of cracks by lowering tensile stresses near the walls of the WAAM alloy [10]. Also, higher travel speeds showed reduced porosity and higher tensile strength [11]. Oscillation of the weld pool minimised the risk of parts getting cracked during WAAM fabrication [12]. Higher interpass temperature reduced the level of porosity in the parts fabricated [13]. A pulsed-AC mode during WAAM fabrication reduced porosity in the part [14]. WAAM-fabricated Al was observed to possess coarser grains along the transverse direction, showed better tensile and ductile properties along the longitudinal direction, and showed consistent hardness values along the longitudinal direction, but showed little variations along the transverse direction [15]. Coarse grains were observed in the upper and middle part of WAAM Al due to heat accumulation and slow heat dissipation, resulting in a decrease in microhardness values [16]. Increasing the wire feed rate from 4 to 8 m/min resulted in an increase in the ultimate tensile strength and elongation. A further increase in the wire feed rate refines the grain size and strengthens the part, which further resulted in reverse trends [17]. WAAM-fabricated Al parts possessed fewer residual stresses and had mechanical properties equivalent to those of the conventional wrought material [18]. Ageing treatment improved the mechanical properties of the part. WAAM-fabricated Al 5356 thin-walled parts showed higher tensile strength when employing the back-and-forth fabrication strategy. Their mechanical properties did not show any correlation with the porosity percentage of the parts [19].

### 1.2. Machinability and Dynamic Behaviour of WAAM-Fabricated Components

The machining of conventional wrought Al alloys showed better surface finish when cutting fluids were employed [20]. Additively fabricated Selective Laser-Melted (SLM) Al alloys showed reduced roughness when machined using higher spindle speeds but the reverse when machined using higher feed and depth of cut [21]. The surface roughness values of the WAAM-fabricated part were observed to be greatly influenced by the feed rate. It was spindle speed in the case of the wrought part [22]. Chatter vibrations were one of the most prominent problems while machining thin-walled components fabricated through WAAM [23]. Higher spindle speeds and moderate feed rates resulted in better surface finish and dimensional accuracy in thin-walled Al parts [24]. Lower feed rates and moderate tool diameters were observed to improve surface finish and reduce cutting forces. Chatter was one of the issues while machining, causing poor surface finish. Higher feed rates were observed to reduce wall thickness deviation [25]. Glue clamping was found to be useful in mitigating wall deformations [26]. High-speed machining of thin-walled Al parts reduced cutting forces and deformation during machining [27]. Increased surface roughness was observed when machined with increased feed rate. A low feed rate, high spindle speed and moderate depth of cut contributed to the optimal cutting conditions that reduced deflection and surface roughness while machining thin-walled Al parts [28]. A review of the existing literature is shown in Table 1, revealing a clear gap in the evaluation of mechanical properties and the machinability of WAAM-fabricated thin-walled aluminium components.

Although Wire Arc Additive Manufacturing (WAAM) has been widely investigated for the production of aluminium alloys, most of the published works have concentrated on process optimisation, defect reduction, microstructural changes and mechanical properties of the fabricated parts. The effect of WAAM process parameters on the formation of porosity and the development of residual stress have been reported previously, along with the influence on the grain morphology and tensile behaviour, with some limited studies reporting on machinability aspects such as cutting forces, surface roughness, tool wear, and dimensional accuracy.

However, there are still some research gaps to be addressed. Firstly, it is not always easy to find direct comparisons of WAAM-produced and conventional wrought aluminium alloys under the same machining conditions, especially for thin-walled structures. The machining behaviour of thin-walled components is not directly applicable to studies on bulk materials, since dynamic instability and vibration-induced errors are very sensitive to the stiffness of thin-walled components. Second, while chatter is known as one of the main factors affecting the productivity and surface quality of thin-walled additively manufactured parts, systematic studies on the chatter susceptibility of WAAM and wrought alloys are still relatively scarce. Specifically, the use of frequency domain analysis of cutting force signals to assess and compare the chatter behaviour of these material conditions has been little investigated. Thirdly, manufacturing-induced microstructural heterogeneity and the machining dynamic response relationship are still not well understood. Few investigations have tried to determine the correlations between material microstructure, mechanical behaviour and chatter-related performance in machining.

To overcome these limitations, the present study compares the performance of WAAM-processed and conventionally processed aluminium alloys during thin-wall milling under similar conditions. Mechanical characterisation, the assessment of surface integrity and frequency domain analysis of cutting force signals are combined to examine the effects of the manufacturing route on machinability and susceptibility to chatter. This is a more complete representation of how material properties and the dynamics of processing interact in thin-walled aluminium components.

Based on the identified gaps, this study addresses the following research questions:

RQ1: How does the WAAM fabrication route influence the mechanical properties (tensile strength, elongation, and hardness) of aluminium alloys relative to conventionally processed counterparts?

RQ2: How does frequency domain analysis of milling force signals reveal differences in dynamic stability and chatter susceptibility between WAAM-fabricated and wrought aluminium specimens?

The objectives of this study are to experimentally investigate mechanical properties and behaviour during the machining of WAAM-fabricated and conventionally processed thin-walled aluminium specimens under similar machining conditions and to analyse their dynamic stability using frequency domain analysis of the cutting force signals.

Knowing the behaviour of WAAM-fabricated thin walls made of aluminium alloys and their effects on mechanical properties and the reactions of processing is essential to ensure successful industrialisation. The novelty of the present work lies in combining both mechanical integrity and dynamic machinability within the same experimental framework. While other studies have focused on metallurgical defects and/or machinability aspects separately, this work uses frequency domain analysis of cutting force signals to study the effect of the microstructural heterogeneity observed in the WAAM process on the dynamics of the stability behaviour during machining. This study offers practical insight into process limitations, machining challenges and potential areas of further optimisation for the practical application of this process in industry by comparing the results to conventionally processed wrought aluminium under identical conditions.

The remainder of this paper is organised as follows: Section 2 presents the research objective, material selected and methodology; Section 3 discusses the results; Section 4 addresses practical implications; and Section 5 concludes with key findings and suggestions for future work.

## 2. Materials and Methods

**Figure 1:**
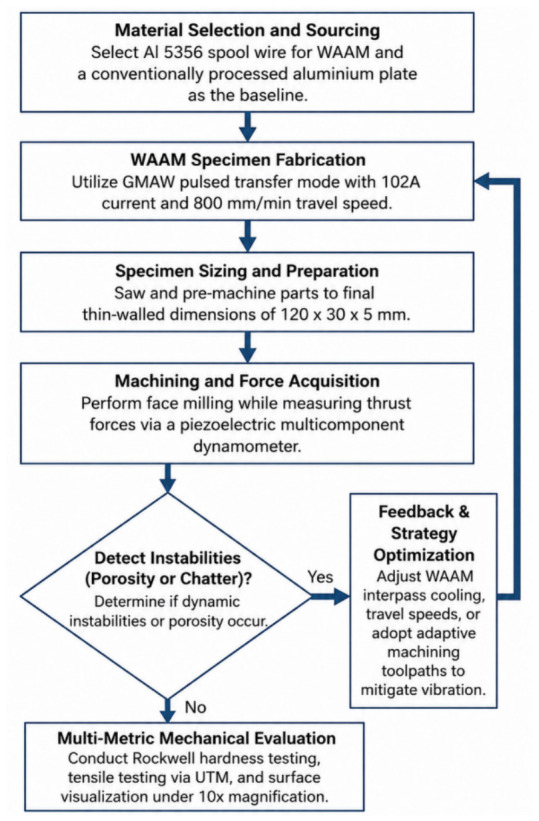
Flowchart representing the experimental workflow.

In the present work, the mechanical properties and the machining response of WAAM-fabricated thin-walled aluminium specimens are experimentally investigated and compared to the properties of their conventionally processed counterparts to understand the effect of the manufacturing route on structural performance and dynamic machinability. To investigate these characteristics systematically, the structure of the Design of Experiments (DOE) was planned, with 20 designed independent milling trials, of which 10 were performed on the conventionally processed sample and 10 on the WAAM-fabricated Al 5356 sample under controlled machine conditions. The experimental methodology is detailed in the following subsections: material selection, WAAM process fabrication, specimen preparation, mechanical setup, mechanical characterisation, and dynamic response analysis. The overall experimental procedure used is shown in Figure 1.

**Figure 2:**
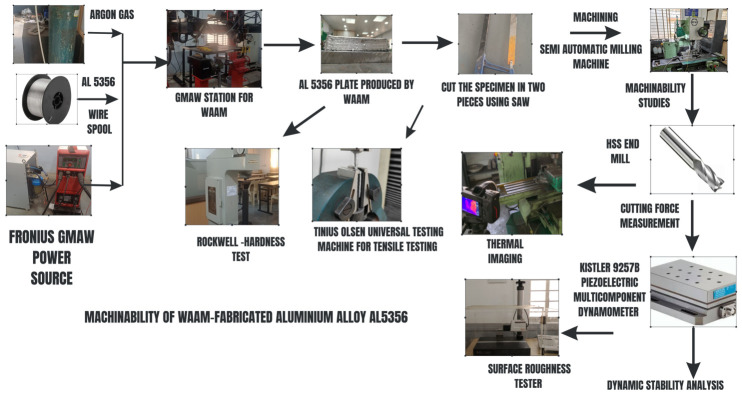
Schematic diagram of process chart.

Figure 2 presents a detailed schematic representation of the experimental methodology adopted in this study, illustrating the complete process sequence from material selection and WAAM fabrication to specimen preparation, controlled milling experiments, dynamic instability assessment through cutting force analysis, thermal characterisation, and mechanical property evaluation. The schematic provides a comprehensive overview of the interrelationship between fabrication route, machining response, and post-machining characterisation employed for the comparative assessment of WAAM-fabricated and conventionally processed aluminium specimens.

### 2.1. Materials

Al 5356 spool wire with a diameter of 1.2 mm was selected for fabricating the WAAM specimen, with deposition carried out on an Al 5083 substrate of dimensions 240 × 75 × 25 mm. The chemical compositions of ER 5356 and ER 5052 are tabulated below in Table 2.

For the comparative evaluation of manufacturing route-induced behaviour, a conventionally processed aluminium specimen was considered as the reference counterpart. Since the focus of this investigation is to assess the influence of manufacturing routes on the mechanical and machining performance of the Al 5356 material system, the comparison is framed in terms of WAAM-fabricated and conventionally processed specimens rather than compositional differences.

### 2.2. WAAM Process Description

**Figure 3:**
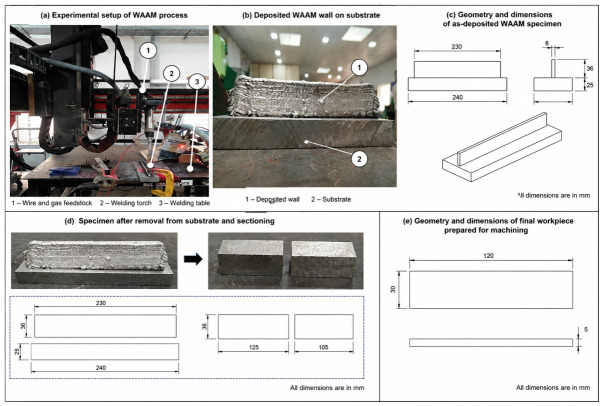
Workflow of WAAM specimen fabrication and machining specimen preparation: (**a**) WAAM experimental setup, (**b**) deposited wall on substrate, (**c**) geometric dimensions of the as-deposited specimen, (**d**) specimen extraction and sectioning from the substrate plate, and (**e**) final workpiece geometry used for mechanical characterisation and machinability investigations.

The WAAM deposition process was carried out using the GMAW process, controlled with a Siemens PLC-based system with an X-Y-Z traverse range of 1000 × 500 × 500 mm, as shown in Figure 3a. Fronius trans Steel 5000 Pulse, operating at 380/400 V and equipped with water cooling, was used as the power source. The WAAM process was carried out with a travel speed of 800 mm/min, shielding gas flow rate of 18 L/min (with pure argon) and a welding current of 102 A. Pulsed transfer mode was chosen as the metal transfer mode for this application. To reduce residual stress and minimise part distortion, 4 min of interpass time was given between the deposition of each layer. The fabricated WAAM specimen shown in Figure 3b, had a thickness of 6 mm, a height of 36 mm and a length of 230 mm, as shown in Figure 3c.

### 2.3. Specimen Preparation and Machining Process

Pre-machining operations were performed to obtain the required workpiece dimension of 120 × 30 × 5 mm after WAAM. The WAAM part was removed from the base plate with the help of a hand saw. The WAAM part was then parted into two sets of dimensions, 125 × 36 × 6 mm and 105 × 36 × 6 mm, as shown in Figure 3d. The 125 × 36 × 6 mm piece was used for machinability studies, whereas the other was used for obtaining the mechanical properties of the WAAM part. The 125 × 36 × 6 mm piece was machined to obtain a piece dimension of 120 × 30 × 5 mm, as shown in Figure 3e.

This step was carried out on a Bharat Fritz Werner Limited (BFW VF-1) manufactured in Bengaluru, India). conventional milling machine featuring a 1000 × 230 mm clamping area, 590 × 270 × 390 mm maximum travel in the X, Y, Z directions, a 45–2000 rpm (12 steps) speed range and a 16–800 mm/min (18 steps) feed range. A high-speed steel (HSS) 6-tooth end mill cutter with 10 mm diameter manufactured by Ru HI was chosen for this application. Similarly, the milling experiments were carried out on conventional aluminium pieces.

**Figure 4:**
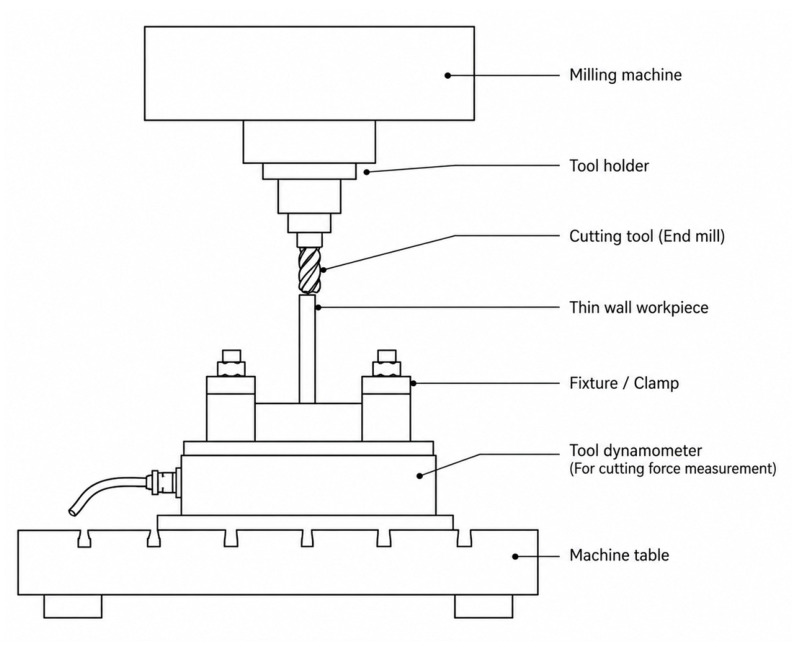
Schematic of the thin-wall milling experimental setup.

To machine the 120 × 30 × 5 mm WAAM part and the 120 × 30 × 5 mm conventional part, the machine table was prepared by setting up the multicomponent dynamometer and its proprietary vise on the vertical milling machine as shown in Figure 4. The specimens were placed vertically with their length (120 mm) becoming their height and their height (30 mm) becoming their length. A height of 45 mm was used for clamping the pieces, leaving out 75 mm as the free height for machining purposes. The height-to-thickness ratio was thus set at 15:1, providing the thin-wall specimen required for machinability studies. Face milling operations were carried out on the pieces as per the cutting parameters set up for experimentation, as shown in Table 3, using a Ru HI End mill cutter with 10mm diameter. While machining, the cutting forces were measured using a Kistler 9257B piezoelectric multicomponent dynamometer (Winterthur, Switzerland) with dimensions 100 × 170 mm. The dynamometer had the capacity to measure forces up to 10 kN. The sampling rate for obtaining the force data was set at 25 kHz. The face milling operations were conducted across a range of spindle speeds and depths of cut while maintaining a constant feed rate, as given in Table 3. Twenty independent milling experiments were conducted according to the cutting parameter matrix presented in Table 3, consisting of 10 trials for the WAAM specimen and 10 trials for the wrought specimen.

A constant feed rate of 80 mm/min was deliberately selected to isolate the microstructural effects on dynamic stability. In thin-walled milling, feed rate is a well-documented primary kinematic driver of chip load. By maintaining a constant feed rate across all trials, the experimental design ensured that any observed variations in thrust force, chatter frequency signatures, and resulting surface degradation could be conclusively attributed to inherent material differences, specifically WAAM-induced porosity and dynamic instability, rather than alterations in cutting kinematics.

## 3. Results and Discussion

All the results obtained on completion of the experimental work, including the power spectrum, tensile strength, elongation (%), and Rockwell hardness, are analysed and discussed in detail in this section.

### 3.1. Tensile Test

A tensile test was conducted using a Tinius Olsen Universal Testing Machine (UTM) (Capacity-25 kN). The specifications include a maximum extension of 600 mm, a speed range of 0.01–500 mm/min and a minimum gauge length of 25 mm. Specifically, the load was applied along the longitudinal direction, in the case of the WAAM specimen, to determine its maximum load-bearing capacity and ductile nature. The pieces were cut using an HSS 30-degree angle milling cutter for this application. The stress–strain values and the stress–strain curves were obtained from the tensile test.

Engineering stress was calculated by dividing the applied load by the original cross-sectional area of the tensile specimen, while engineering strain was determined using the specimen gauge length in accordance with ASTM E8/E8M standards [29]. The stress–strain curves presented in this study were generated from the measured gauge length deformation data obtained during testing. Particular care was taken to ensure that the reported tensile strength and elongation values were derived from the specimen deformation response rather than machine compliance effects. Since the primary objective of the tensile characterisation was to compare the strength and ductility of the WAAM-fabricated and conventionally processed specimens, the discussion focuses on ultimate tensile strength and total elongation.

**Figure 5:**
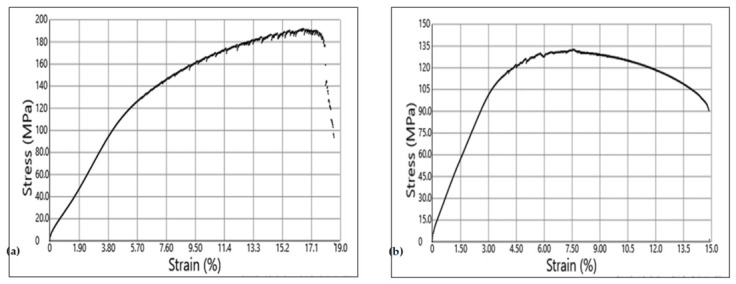
Stress–strain curves obtained from tensile testing of (**a**) WAAM-fabricated Al 5356 specimen and (**b**) conventionally processed wrought aluminium specimen.

**Figure 6:**
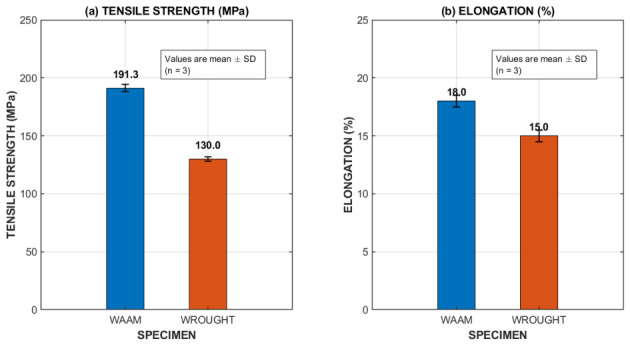
Comparison of mechanical properties of WAAM-fabricated and wrought specimens showing mean values with ±1 standard deviation error bars: (**a**) tensile strength and (**b**) elongation percentage.

Tensile testing revealed that the WAAM specimens achieved an ultimate tensile strength (UTS) of 192 MPa, demonstrating a 44.36% enhancement over their wrought counterparts. Concurrently, the WAAM parts exhibited superior ductility, attaining a total elongation of 18.2% compared to the 14.9% observed in the wrought baseline. The observed increase in tensile strength and elongation could be attributed to an interaction of the alloy composition, the thermal history of the WAAM process and the microstructural evolution reported in previous publications. Because this directional microstructure aligns parallel to the principal loading axis during testing, it effectively maximises the material’s load-bearing capacity and facilitates greater plastic deformation before fracture. These results align with the studies reported in the literature. The stress vs. strain (%) curves for both specimens are shown in Figure 5a. Figure 5b compares the tensile strength of both specimens, and Figure 6a,b compares the elongation percentage of both the specimens. It should be noted that the filler wire used to fabricate the WAAM specimen is ER5356, whereas the conventional processed reference specimen is the composition of Al5052 alloy. Hence, the observed variations in mechanical properties are the result of alloy compositions and processing. In the present study, comparative behaviour is examined instead of an isolated assessment of manufacturing route effects, under identical machining conditions.

### 3.2. Hardness Test

The Rockwell hardness test reported an HRB of 63 for the WAAM part and an HRB of 68 for the wrought part. The WAAM part was observed to be a little softer than the wrought part. The observed 7.35% reduction in Rockwell hardness (63 HRB vs. 68 HRB) in WAAM components stems from the intrinsic ‘in situ’ annealing effect. Hardness measurements were obtained from three different locations on each specimen, and the reported values represent the average of these measurements.

**Figure 7:**
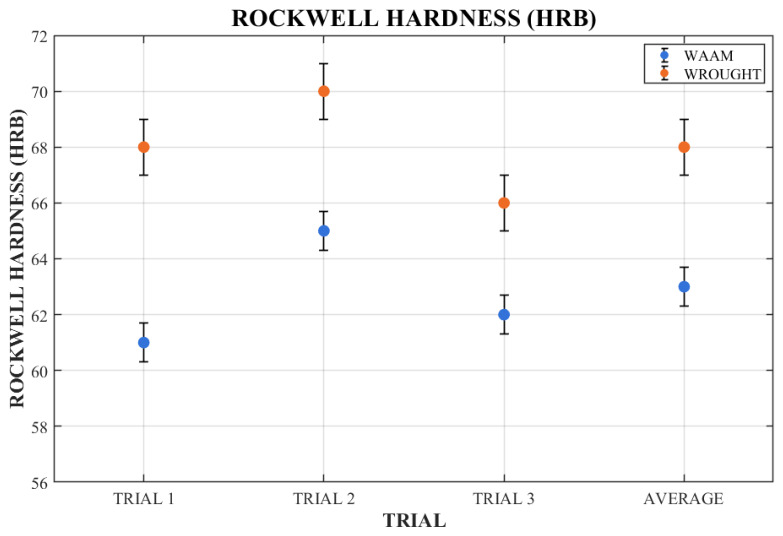
Comparison of Rockwell hardness test results.

The repeated thermal cycling from subsequent deposition layers facilitates grain growth and reduces dislocation density, thus effectively softening the matrix relative to the strain-hardened wrought state (Köhler et al., [17]; Sharma et al., [16]). The Rockwell hardness test results are summarised in the plot comparing the Rockwell hardness values of both the specimens. The hardness values presented in Figure 7 are expressed as mean values with standard deviation error bars.

### 3.3. Dynamic Stability Analysis

The comparative dynamic analysis of WAAM and wrought aluminium plate specimens, carried out during the vertical milling process, reveals that the two have different susceptibilities to chatter, and the WAAM specimen exhibits better dynamic stability at the same machining conditions. The spindle speed was fixed at 1000 rpm, the feed rate at 80 mm/min, and the axial depth of cut at 1.5 mm in the experiments. The relatively deeper depth of cut was chosen as a severe cutting condition to increase the chance of chatter being excited and to provide a more suitable basis for the comparison of chatter resistance. The same patterns in responses were seen for the other combinations of machining parameters, thus demonstrating the repeatability of the observed behaviour. The feed force time domain response was measured during the cutting process with a Kistler tool dynamometer and subsequently analysed in the frequency domain and time–frequency domain to evaluate the cutters’ tendency to chatter.

**Figure 8:**
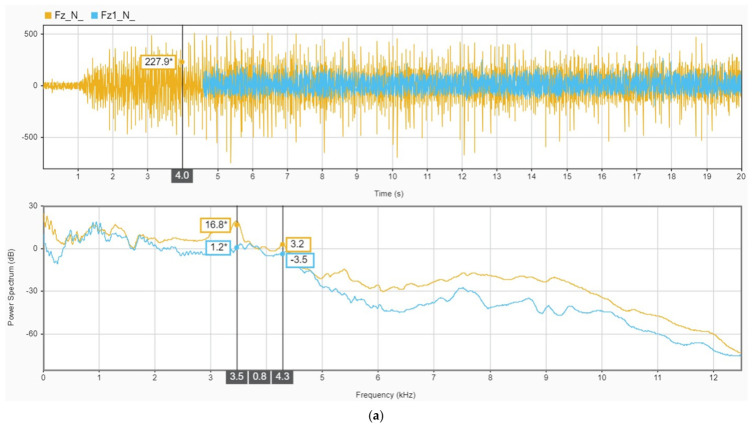

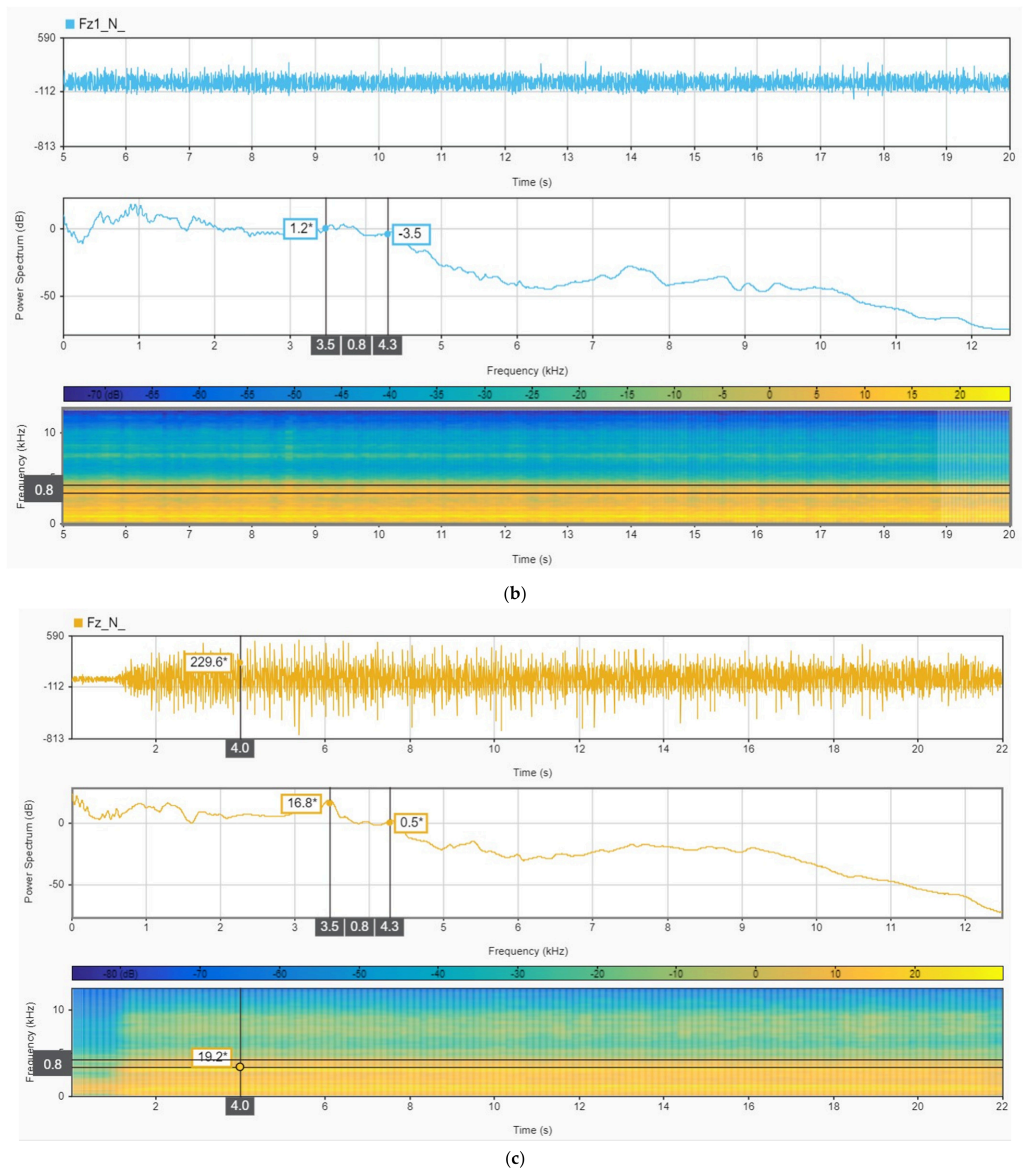
(**a**) Dynamic feed force analysis: power spectrum of the cutting force signal. (**b**) Dynamic feed force analysis: spectrogram of the wrought aluminium specimen. (**c**) Dynamic feed force analysis: spectrogram of the WAAM−fabricated aluminium specimen. (*) indicates the peak response.

The power spectrum reveals, as shown in Figure 8a–c, two dominant dynamic peaks for both materials. The first peak is at about 3500 Hz, which is the experimentally determined natural frequency of both the plate specimens, showing that the milling excitation is very effective in activating the dominant structural mode. A second sharp peak is observed at ~4300 Hz, suggesting that another structural or chatter-related dynamic mode is excited. Importantly, the resonant amplitude of this WAAM specimen is significantly lower than that of the wrought aluminium specimen at both resonant peaks, demonstrating less vibration amplification for the same vibration excitation. The reduction in the spectral amplitudes for WAAM suggests that the process has greater resistance to chatter onset, as this is closely linked with the amplitude of resonance and the growth of the regenerative forces.

The wrought aluminium specimen has high broadband spectral energy over the entire frequency range analysed, suggesting high force modulation and continued dynamic amplification of the signal beyond those of the dominant peaks. The WAAM specimen, by contrast, exhibits consistently lower magnitudes in the spectrum, suggesting that this would lead to stronger suppression of vibration growth and weaker regenerative excitation. The spectrogram analysis also corroborates this conclusion. The wrought aluminium sample shows continuous high broadband energy bands throughout the entire machining process, which is a typical characteristic of a chatter-prone cutting with sustained self-excited vibration. The WAAM specimen, by contrast, exhibits much lower levels of the energetic bands’ intensity, narrower frequency occupation and lower temporal persistence of energetic bands, which suggests a more stable cutting response.

The material properties inherent in the WAAM process could have contributed to the better chatter resistance of the WAAM specimen. The layer-wise deposition mechanism forms a heterogeneous grain morphology, interlayer metallurgical interfaces, a redistribution of residual stress, and localised microstructural discontinuities, which can contribute to an increase in the internal energy dissipation and effective damping under dynamic excitation. This increased damping ability can help to reduce the danger of resonance amplification and delay the onset of regenerative chatter during multiple tooth engagement in vertical milling. Based on the combined power spectrum and spectrogram analysis of feed force data acquired from the Kistler, it can be concluded that the WAAM aluminium specimen has better dynamic stability and chatter initiation and propagation resistance than the wrought aluminium specimen.

### 3.4. Influence of Material Condition on Surface Roughness

**Figure 9:**
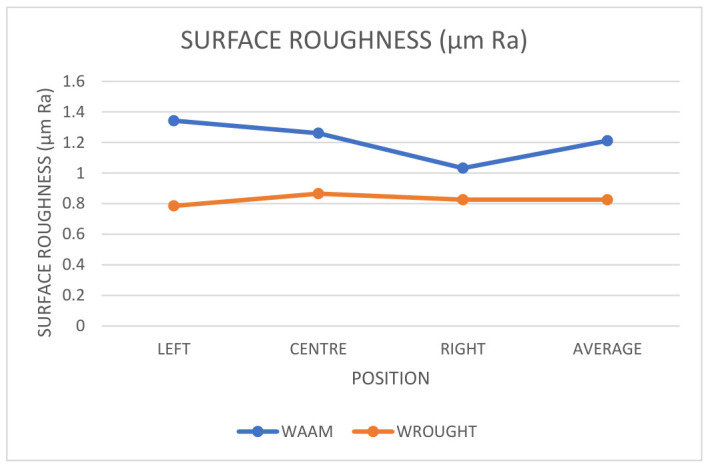
Comparison of surface roughness test results.

The surface roughness tested using Mitutoyo (SJ-410) column-type surface roughness tester (Swidnica, Poland), of the fabricated alloy was measured using the same locations in the two alloys; as seen in Figure 9, the WAAM-fabricated alloy had a consistently higher roughness than the wrought alloy. The average roughness of the WAAM specimen was 1.22 μm Ra, while, for the wrought alloy, it was 0.83 μm Ra. Roughness was greatest for the WAAM specimen at the left position (1.35 μm Ra), while it was relatively similar for the wrought specimen (0.79–0.87 μm Ra). Though the frequency domain analysis showed that the WAAM alloy had better vibration stability, the surface finish results showed that the machined surface quality was not necessarily determined by the vibration stability. The chatter resistance is mainly determined by the capability of the material–tool system to dampen the self-excited vibrations, and the surface roughness is further affected by the material microstructure, hardness distribution, porosity and local cutting mechanics.

The WAAM process includes the deposition of the metal layers by layer and also repeated thermal cycles, which create heterogeneous microstructural regions, interlayer boundaries and localised defects. These features may result in non-uniform plastic deformation and irregular chip formation during machining, which may cause increased surface asperities. Therefore, although the vibration amplitudes were found to be low and the dynamic stability was found to be good for the WAAM alloy, the roughness values were higher due to the heterogeneous material. The wrought alloy, on the other hand, has a more uniform microstructure and mechanical behaviour, leading to uniform material removal and surface generation. Thus, the lower roughness value of the wrought alloy is mainly due to its microstructural uniformity and not to vibration behaviour.

### 3.5. Machining Mechanics and Thermal Behaviour

**Figure 10:**
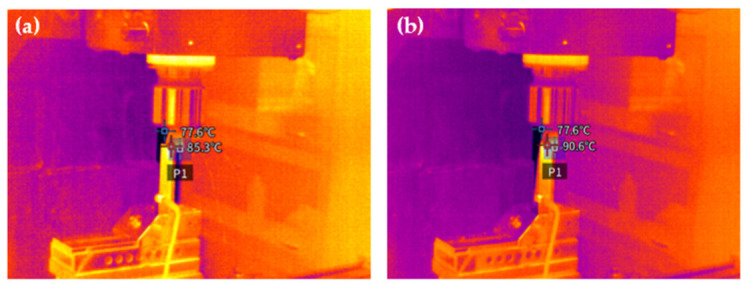
Thermal profiles extracted from infrared measurements during machining: (**a**) conventionally processed wrought specimen and (**b**) WAAM-fabricated specimen.

**Figure 11:**
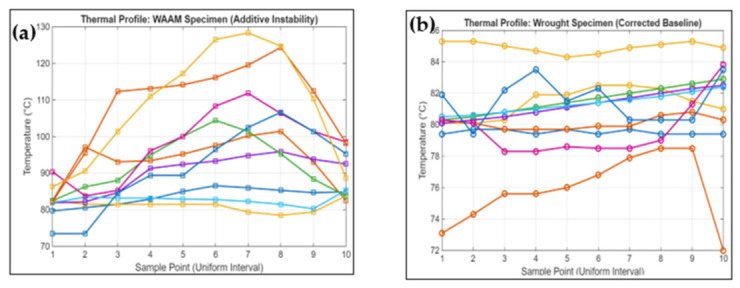
Comparison of thermal profiles during machining for (**a**) the WAAM-fabricated specimen and (**b**) the wrought specimen.

For comparison, the Al 5356 material system fabricated using WAAM was milled under the same machining conditions as the conventionally processed Al 5356, and the thermal response of the system was recorded using an a Sonel KT-560 infrared thermal imaging camera (Poland). The thermal distributions in the infrared images shown in Figure 10a,b clearly show a difference in the heat generation behaviour depending on the manufacturing route. The conventionally processed specimen had a relatively lower temperature distribution that was more uniform in the cutting zone compared to the WAAM-fabricated specimen, which had a noticeably higher localised thermal concentration in the interaction between the tool and the workpiece. This is also evident in the corresponding thermal profiles displayed in Figure 11.

The temperature range of the wrought sample was also relatively stable compared to the relatively large variations measured along the sample, and was comparatively smaller compared with the other sampled points, which suggests a more uniform thermal response during the process. The temperature peaks and levels recorded were, however, significantly higher in the WAAM sample, and the thermal variation between the measurement points was much larger, with the peak values being considerably higher than the wrought sample. This is a sign of less stable material removal behaviour. This is because the microstructural properties of the WAAM specimen are not homogeneous, and the tensile strength is higher than that of the other specimens.

The WAAM material has more resistance to deformation, which means that higher cutting energy is needed for machining and, consequently, higher cutting temperature will be generated at the tool–workpiece interface. Furthermore, interlayer metallurgical interfaces, localised non-uniformities in the microstructures, and internal discontinuities can also help create non-uniform heat dissipation, which helps lead to localised thermal accumulation. The greater temperature variations found in the WAAM specimen shown in Figure 11 also indicate that the cutting process in this material is not as uniform as the wrought material. This thermal instability can occur together with varying cutting forces and periodic dynamic actions between the tool and the workpiece. Thus, the thermal analysis suggests that the WAAM-fabricated aluminium will have a more thermally demanding and non-uniform machining response in comparison to the conventional wrought aluminium under the set cutting conditions.

## 4. Discussion Section

The experimental findings indicated significant variations in mechanical properties and in dynamic behaviour in the machining of the WAAM-fabricated and wrought aluminium test pieces. The tensile strength of the WAAM specimen was 192 MPa, while that of the wrought specimen was 133 MPa, which is an improvement of 44.36%. Likewise, the elongation of the wrought specimen was 14.9%, while the WAAM specimen was 18.2%, showing that the latter is more ductile. But the WAAM sample had somewhat smaller hardness values than the wrought sample. The mechanical properties are summarised in a comparative table (Table 4).

### 4.1. Response to RQ1: Influence of WAAM Fabrication Route on Mechanical Properties

The first research question focused on whether the mechanical properties of aluminium alloys are affected by the WAAM fabrication route compared with those of wrought alloys. The results show that the WAAM fabrication route improves the tensile performance and slightly decreases hardness. In particular, the WAAM specimen had a tensile strength that was 44.36% better than that of the wrought specimen, and an elongation that was 22.15% higher, suggesting that it was a better load-bearing material and more ductile. This improvement is partly due to the layer-wise deposition process, which creates a directional microstructural feature. In WAAM fabrication, the repeated melting and solidification cycles during the operation tend to cause the grain structure to align in the deposition direction, resulting in this anisotropic grain alignment. In the present study, tensile loading was applied in the longitudinal direction of the WAAM specimen and was thought to have had a positive effect on load transfer efficiency and on the extent of plastic deformation before failure. In addition, the repeated thermal cycling during deposition might allow for some redistribution of stress at the local level that could help to increase ductility. Even though the tensile behaviour improved, the WAAM specimen had a moderate decrease in hardness compared with the wrought material. This behaviour is like the thermal histories that result from additive deposition, where multiple reheating during successive layers may create an in situ annealing effect that can lead to grain coarsening, lower dislocation density, and thus soften the matrix material.

For this reason, the WAAM fabrication route was seen to increase tensile strength and ductility compared to the wrought fabrication route, but with a slight decrease in hardness due to thermal softening effects, which can be seen as a response to RQ1.

### 4.2. Response to RQ2: Frequency Domain Dynamic Stability Behaviour

The second research question is about the use of frequency domain analysis on milling force signals to identify the differences in dynamic stability behaviour between WAAM-fabricated and wrought aluminium specimens. The power spectral analysis of cutting force signals showed that there were two major frequency peaks in both materials at around 3.5 kHz and 4.3 kHz. The first peak is the main natural frequency occurring in the structure when it is excited during milling, and the second peak represents a higher dynamic mode that is excited due to the vibration behaviour when the structure is cut during milling. The important point to be noted from the power spectrum is that the amplitude of the spectral components of the WAAM specimen is much lower than that of the wrought specimen for both dominant frequencies. The lower the spectral amplitude is, the lower the resonance amplification will be in the same machining excitation condition, which means that the dynamic stability in the milling process will be higher. This is important because the reduced spectral response in the WAAM specimen suggests that it has a relatively lower susceptibility to the growth of unstable vibrations, which are closely linked with regenerative chatter. A comparison of the frequency domain response again reveals that the wrought sample had significantly greater broadband spectral energy in the frequency range analysed, signifying more force fluctuations and continued dynamic excitation. The WAAM specimen, on the other hand, had consistently lower broadband spectral magnitudes, suggesting lower force modulation and a higher attenuation of vibration propagation. This interpretation is confirmed by the spectrogram analysis. The wrought aluminium specimen exhibited continuous high-intensity frequency bands throughout the machining time, indicating a sustained self-excited vibration behaviour. On the other hand, the WAAM specimen showed lower spectral intensity, lower temporal persistence, and a narrower frequency occupation, showing a stable cutting response under the same cutting conditions. This increase in dynamic stability for the WAAM specimen can be explained by the heterogeneities of its microstructural characteristics created in the process used for WAAM. All of the above can combine to provide improved internal damping properties, which in turn will minimise resonance magnification in repeated tooth contact.

Therefore, for RQ2, the frequency domain analysis showed that the WAAM-fabricated sample had lower vibration amplification, lower broadband spectral energy and higher dynamic stability than the wrought sample, suggesting a higher resistance to dynamic instability under the cutting conditions investigated due to the machining process.

## 5. Practical Implications

The high tensile strength and elongation of WAAM-manufactured Al alloys allows for the production of lighter and thinner parts. This ability enables better structural performance, material savings, and enhanced fuel efficiency. WAAM’s ability to manufacture parts close to the final desired shape improves material yield and reduces lead times, which makes it ideally suited for customised manufacturing and rapid prototyping.

The high roughness of the surface and high chatter tendency make the machining process a challenge. Conventional parameters may be insufficient for controlling surface and dimensional accuracy of the workpiece; thus, selected spindle speeds, adaptive toolpaths and better tools are necessary. Additionally, some parts may need to be polished and/or vibrated to achieve the desired surface finish. The installation of monitoring systems which can detect chatter when the machine is operating can help to significantly improve stability in the cutting process.

## 6. Conclusions

For the successful integration of Wire Arc Additive Manufacturing (WAAM) in high-precision industries, it is important to understand how the part’s internal structure influences its behaviour when the part is subjected to the cutting tool. This study bridges the gap between the porosity generated during fabrication and dynamic chatter, establishing the actual cost of the ‘machinability penalty’ of AM. The main results, contributions, and future recommendations of this study are as follows:

### 6.1. Key Findings

The main conclusions of this work show that there is a main conflict between structural integrity and machinability in thin-walled WAAM structures. The additive process showed a significant increase in bulk tensile strength (44.36%) due to the alignment of the grains along the build direction, but the process created a negative impact on the material’s machinability. This penalty was manifested by a 46.73% rise in the average surface roughness. Moreover, it was found that subsurface porosity is a catalyst to induce dynamic instability in the process of machining. The instability was seen under the tested geometric and cutting conditions as a separate chatter peak at 970 Hz. Finally, such frequency domain instabilities are directly responsible for the surface degradation observed, as a permanent ‘chatter mark’ is left throughout the surface by the high-frequency oscillations of the tool.

### 6.2. Contributions

One of the key scientific findings of this research is that a quantitative relationship between frequency domain analysis and dynamic machining instability with regard to subsurface porosity, a defect that occurs in the process of additive fabrication, has been successfully established. The study makes this link, fundamentally challenging the current manufacturing standards. It states that traditional machining rules, which are optimised for uniform wrought alloys, cannot be directly applied to additively manufactured components. The non-homogeneous internal structures of WAAM parts can trigger self-excited vibrations, thus necessitating a defect-specific machining approach.

### 6.3. Limitations and Future Work

The outcomes of this study depend on the experimental conditions and the setup used for machining in the experiments. The measured chatter frequency and machining responses can be different at other operating conditions. Furthermore, the conventionally processed reference specimen was chosen as a reference sample for comparison with the WAAM-fabricated Al 5356 sample due to the similar characteristics of the Al–Mg alloy and metallurgical behaviours. However, slight changes in composition could affect some mechanical properties.

Future research can be directed towards optimising the WAAM deposition and machining parameters to further enhance the uniformity of the microstructural properties, surface finishing and machining stability. Thin-walled aluminium parts made through WAAM can be further improved for industrial applications by using advanced monitoring techniques and defect-aware machining strategies.

## Figures and Tables

**Table 1:** Summary of key Internet-based references.

Theme	Key References	Relevance to RQs	Contributions & Insights	Identified Gaps
1.1 Context & Applications	Bolar et al. [1]; Sarıkaya et al. [2]	Baseline Context	Defines thin-walled structures (3–5 mm) and establishes their necessity in aerospace and marine sectors due to high corrosion resistance and strength-to-weight ratios.	Lack of direct comparative data between AM-fabricated and wrought thin-walled structures in real-world loading conditions.
1.2 WAAM of Aluminium	Suryakumar et al. [3]; Wu et al. [4]	Baseline Context	Identifies WAAM as a superior AM method for large-scale parts, offering 40–60% reduction in fabrication time and high deposition rates (15–130 g/min).	Research focuses heavily on deposition efficiency rather than the secondary processing (machining) required for functional use.
1.3 Metallurgical & Mechanical Challenges	Zhao et al. [5]; Horgar et al. [7]; Vimal et al. [12]; Aldalur et al. [14]; Bellamkonda et al. [15]; Sharma et al. [16]; Köhler et al. [17]	RQ1	Documents inherent defects like porosity (gas turbulence) and coarse grains (heat accumulation). Proposes interpass cooling and pulsed-AC modes to refine the microstructure and improve tensile properties.	Studies often treat metallurgical refinement in isolation, without investigating how these grain structures behave under cutting forces.
1.4 Machinability of AM Components	He et al. [21]; Yan et al. [22]; Chernovol et al. [23]; Lukic et al. [24]; Borojevic et al. [25]; Zawada-Michałowska et al. [27]; Ramanaiah et al. [28]	RQ2	Highlights chatter and wall deformation as primary failures in thin-walled machining. Establishes that feed rate is the dominant factor for surface roughness (Ra) in WAAM parts.	Absence of a comparative performance baseline for dynamic force signatures (PSD analysis) between WAAM and wrought aluminium.

**Table 2:** Comparative chemical composition (wt%) of ER 5356 and ER 5052.

Elements	ER 5356-Weight (%)	ER 5052-Weight (%)
silicon	0.25	0.25
copper	0.10	0.10
iron	0.40	0.40
manganese	0.20	0.10
magnesium	5.50	2.50
zinc	0.10	0.10
chromium	0.20	0.25
titanium	0.20	-
aluminium	92.90	96.15
others	0.15	0.15

**Table 3:** Experimental cutting parameters utilised for face milling.

Speed (rpm)	Depth of Cut (mm)	Feed Rate (mm/min)
1000	0.50	80
1000	0.75	80
1000	1.00	80
1000	1.25	80
1000	1.50	80
1400	0.50	80
1400	0.75	80
1400	1.00	80
1400	1.25	80
1400	1.50	80

**Table 4:** Comparative mechanical and machining properties.

Property	WAAM	Wrought	Difference
Tensile Strength (MPa)	192	133	+44.36%
Elongation (%)	18.2	14.9	+22.15%
Hardness (HRB)	63 ± 2.08	68 ± 2.08	−7.35%

## Data Availability

The original contributions presented in this study are included in the article. Further inquiries can be directed to the corresponding authors.

## Abbreviations

The following abbreviations are used in this manuscript:

WAAM	Wire Arc Additive Manufacturing
AM	Additive Manufacturing
GMAW	Gas Metal Arc Welding
GTAW	Gas Tungsten Arc Welding
DOE	Design of Experiments
PSD	Power Spectral Density
UTM	Universal Testing Machine
UTS	Ultimate Tensile Strength
HRB	Rockwell Hardness Scale B
IR	Infrared
